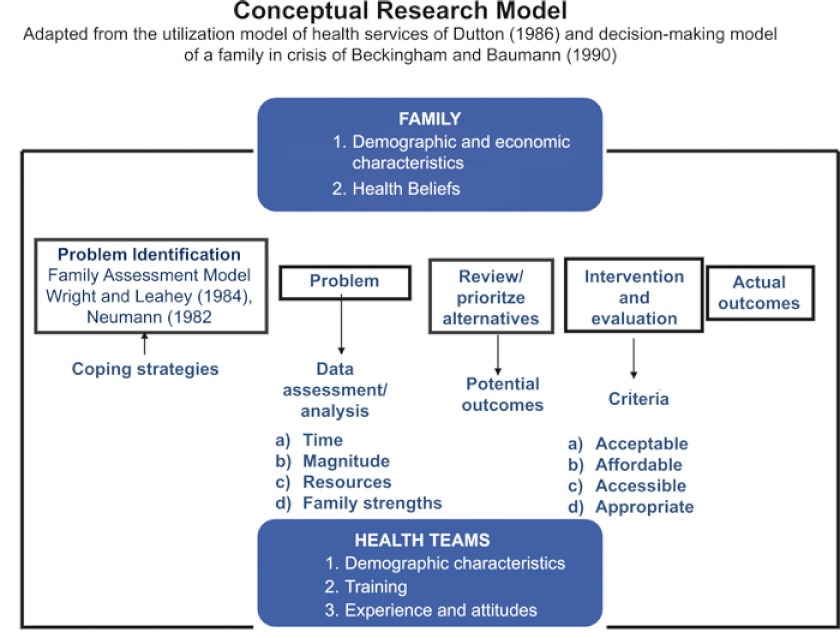# Transferring patients to tertiary health care: the role of family and Primary Health Care Teams

**Published:** 2012-09-04

**Authors:** Filipa Joaquim, Luís Lapão

**Affiliations:** Instituto de Higiene e Medicina Tropical, Universidade Nova de Lisboa, Portugal; Instituto de Higiene e Medicina Tropical, Universidade Nova de Lisboa, Portugal

**Keywords:** integration competences, integrated care, health care delivery, primary health care, governance

## Abstract

**Purpose:**

One of the main problems of health systems is to ensure access and proper coordination between different health services. This problem is particularly felt when a patient is transferred from one level to another, especially for long-term care.

Therefore, it is essential to develop new organizational models, which should meet user’s need. The primary Health Care (PHC) within the current Portuguese Primary Health Care Reform (PPHCR) could develop an important role, due to intrinsic innovative aspects associated with new governance model based on the development of a small family health multi-professional unit (USF) with functional and technical autonomy, integrated within a network with other functional units and the establishment of Health Center Clusters, producing meaningful healthcare delivered value.

**Theory:**

Grounded on the health services utilization model of Dutton (1986) and on multidisciplinary assessment and decision-making model of Beckingham and Baumann (1990) for ageing families.

**Research question:**

In the PPHCR context, how families experience the decision-making process of transferring an elderly relative with dementia?

**Research method:**

An exploratory case study, with two embedded units of analysis (Ying 2003): families of the elderly with dementia and primary health care teams, Data were collected through semi-structured interviews and focus group. The data analysis was achieved through thematic content analysis (Bardin 2008) and descriptive statistical analysis with the support of computer program SPSS. See Figure 1 for the conceptual research model.

**Results:**

Families who had shared their decision-making process with health care teams, recognize their fundamental role as mediator between the families and the complexity of the health and social system. The families, who had been followed up by the Primary Health Care Teams (PHCT), were more satisfied with their decision. However, there still exist some difficulties to address in order to benefit from a more integrated care:

- The communication between the different functional health units and health and social professionals is sometimes inadequate, with gaps, causing delays in response time, especially in crisis situations;
- Misunderstood and overlap competencies among team members;
- Different interpretations of referrals practices and clinical criteria to transferring patients.
- Restrict use of new technologies.

**Conclusions:**

The PPHCR has been an opportunity to create innovation in healthcare delivering value, which is recognized through the families who had been accompanied by the Primary Health Care Teams. However, there still exist some problems, mainly associated to team-work, communication and the use of new technologies. To achieve an innovation culture of excellence it is fundamental the development by PHCT of integration competences and the capacity to introduce project-based approaches that would foster collaboration initiatives.

**Discussion:**

- What are the key integration competences that PHCT must domain?
- How can we measured the degree of health care delivery integration?

## Figures and Tables

**Figure:**